# ATG9A supports *Chlamydia trachomatis* infection via autophagy-independent mechanisms

**DOI:** 10.1128/spectrum.02774-23

**Published:** 2023-09-14

**Authors:** Michitaka Suzuki, Tomoko Funakoshi, Keigo Kumagai, Masaaki Komatsu, Satoshi Waguri

**Affiliations:** 1 Department of Anatomy and Histology, Fukushima Medical University School of Medicine, Hikarigaoka, Fukushima, Japan; 2 Department of Physiology, Juntendo University Graduate School of Medicine, Bunkyo-ku, Tokyo, Japan; 3 Department of Biochemistry and Cell Biology, National Institute of Infectious Diseases, Toyama, Shinjuku-ku, Tokyo, Japan; LSU Health New Orleans, New Orleans, Louisiana, USA

**Keywords:** ATG9A, *Chlamydia trachomatis*, autophagy, clathrin adapter protein

## Abstract

*Chlamydia trachomatis* infection can be regulated by autophagy-related (*ATG*) genes. Here, we found that the depletion of *ATG9A*, one of the core *ATG* genes, in HeLa cells suppressed *C. trachomatis* growth in the inclusion. The growth was restored by re-expressing *ATG9A* or an *ATG9A* mutant impairing lipid scramblase activity in *ATG9A*-knockout (KO) cells. Moreover, the depletion of lipid transfer proteins ATG2A/B, responsible for isolation membrane expansion together with ATG9A, did not significantly alter the growth, suggesting that the non-autophagic function of ATG9A supports *C. trachomatis* infection. *ATG9A*-KO cells showed no infection-induced redistribution of the Golgi from the perinuclear region to inclusion, which was restored by re-expressing the mutant but not the *ATG9A* mutant lacking an N-terminal adapter protein-binding domain. Re-expression of the N-terminal deletion mutant in *ATG9A*-KO cells did not rescue *C. trachomatis* growth, suggesting the importance of this domain for its growth. Although *ATG9A*-KO cells showed enhanced TBK1 activation, interferon (IFN)-β was not significantly increased, excluding the possibility that upregulation of stimulator of IFN genes (STING) signaling suppressed bacterial growth. Taken together, these findings suggest that the proper trafficking, rather than the isolation membrane expansion function, of ATG9A assists *C. trachomatis* growth in the inclusion.

**IMPORTANCE:**

*ATG9A* is an autophagy-related gene that functions during the isolation membrane expansion process to form autophagosomes, but it also has other functions independent of autophagy. In this study, we employed *ATG9A*-deficient HeLa cells and found that the absence of *ATG9A* negatively impacted proliferation of *Chlamydia trachomatis* in inclusions. Furthermore, rescue experiments using *ATG9A* mutants revealed that this action was mediated not by its autophagic function but by its binding ability to clathrin adapter proteins. These findings suggest that the proper trafficking of ATG9A assists *C. trachomatis* growth in the inclusion.

## INTRODUCTION

*Chlamydia trachomatis* is an obligate intracellular gram-negative bacterium that causes sexually transmitted urogenital infections and non-congenital blindness. Its developmental cycle is characterized by the presence of two metabolically distinct forms: a relatively inactive elemental body (EB) and an active reticulate body (RB). The infection begins with the attachment of EBs to the plasma membrane of host cells. EBs are then internalized into the cells and form a membrane-bound vacuole called the chlamydial inclusion. In the inclusion, EBs are converted to RBs that replicate for proliferation and undergo secondary differentiation to EBs through intermediate bodies (IBs). Proliferated EBs are then released from the host cell through lysis or extrusion (1 – 3). During this developmental cycle, *Chlamydia* secretes several effector proteins into the host cell cytoplasm through the type III secretion system to facilitate chlamydial invasion and proliferation. *C. trachomatis* also acquires nutrients including amino acids, nucleotides, and lipids from the host cell through multivesicular bodies, lipid droplets (LDs), mitochondria, endoplasmic reticulum (ER), and Golgi-derived vesicles. For example, sphingomyelin (SM) is synthesized from ceramide by sphingomyelin synthase 1 (SMS1) and SMS2 localizing at the Golgi and/or plasma membrane (4). Under *C. trachomatis* infection, the inclusion acquires SM by intercepting Golgi-derived vesicles transported to the basolateral surface of polarized cells (5, 6). Moreover, the chlamydial deubiquitinase Cdu1 converts normal Golgi architecture into mini Golgi stacks and relocates them around the perimeter of the inclusion, which is thought to facilitate SM supply to *Chlamydia* (7, 8). In fact, preventing Golgi relocation by expressing golgin-84 mutant (N-terminal deletion mutant) or caspase inhibitor Z-WEHD-FMK treatment decreased SM supply and inhibited *Chlamydia* growth (9).

Host cells are equipped with innate immune systems including autophagy to protect them from bacterial infection. During conventional macroautophagy, the isolation membrane (or phagophore) is formed and expands in the cytoplasm, sequestering a portion of cytoplasmic components including organelles and bacteria. When the isolation membrane closes, it becomes an autophagosome, which then acquires lysosomal enzymes by fusing with lysosomes to degrade its contents. This process is regulated at the molecular level by autophagy-related (*ATG*) genes. The formation of the isolation membrane is initiated by the ULK1 complex (including ULK1, FIP200, ATG13, and ATG101), which recruits ATG9A vesicles and class III PI3K complex (including VPS34, Beclin l, ATG14, p115, and AMBRA1) for local production of phosphatidylinositol-3-phosphate (PI3P) at the ER. Then, WIPI2 (a PI3P-binding protein) and ATG2 (a lipid transfer protein) are recruited to this site. ATG2 together with ATG9A (a lipid scramblase) provides a lipid-supplying system for isolation membrane expansion. The ATG12–ATG5–ATG16L complex is also recruited to this site to enhance ATG3-mediated conjugation of ATG8 family proteins including LC3 with phosphatidylethanolamine, which can interact with autophagy receptors including SQSTM1/p62 (10 – 12). Some investigations have reported that *ATG5* deficiency promotes *C. trachomatis* infection (13, 14), while other studies have not reported any effect of either *ATG5* or *ATG7* deficiency on the infectious process (15, 16). Therefore, the effect of autophagy suppression on chlamydial infection remains controversial. However, the microtubule-dependent association of LC3 with the chlamydial inclusion is essential for *C. trachomatis* infection, which is thought to be due to autophagy-independent functions (13). A recent study revealed that ATG16L is targeted by *Chlamydia* effector protein TaiP (CT622), which disrupts ATG16L1–TMEM59 interaction, rerouting Rab6-positive vesicles toward the inclusion. This is also independent of the autophagic function of ATG16L (17).

Although ATG9A is one of the factors initiating autophagy induction, it also has autophagy-independent functions, including membrane-bending ability (18); fatty acid mobilization from LDs to mitochondria (19); promotion of HIV infectivity (20); protection from plasma membrane permeabilization (21); and regulation of stimulator of interferon genes (STING) signaling (22), JNK signaling (23), and actin cytoskeleton (24). Since *C. trachomatis* acquires nutrients from the post-Golgi compartments and ATG9A vesicles move between the Golgi, endosomes, and plasma membrane, ATG9A trafficking may contribute to *C. trachomatis* growth. Therefore, we investigated the roles of ATG9A during *C. trachomatis* infection using *ATG9A*-knockout (KO) HeLa cells. We found that ATG9A supports *C. trachomatis* growth in the inclusion. Interestingly, the supportive effect of ATG9A is not mediated by autophagy, but by its adapter protein (AP)-dependent trafficking between the post-Golgi compartments.

## RESULTS

### ATG9A supports *C. trachomatis* growth in the inclusion

**Fig 1:**
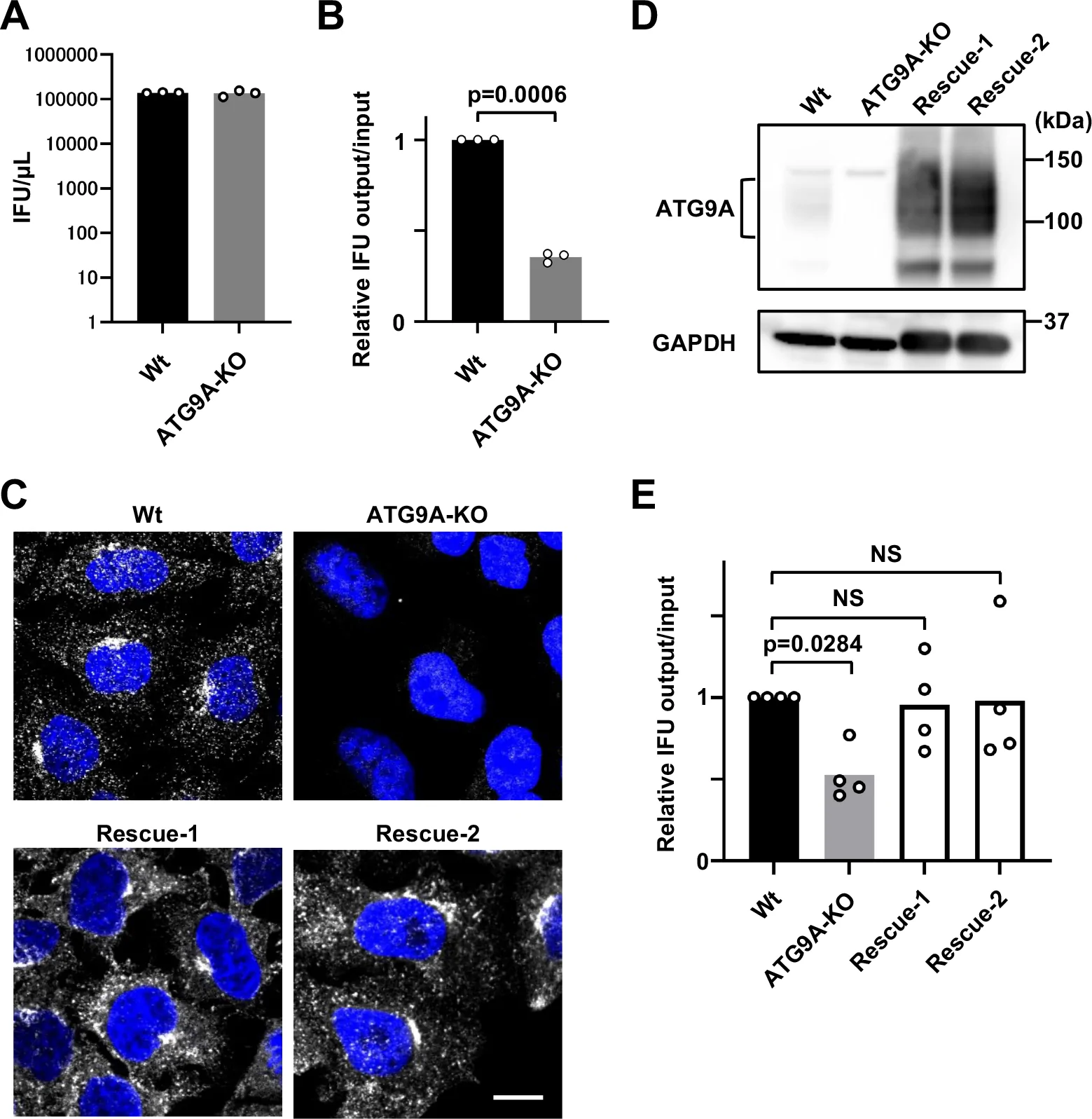
ATG9A supports *C. trachomatis* growth in the inclusion. (**A**) Inclusion-forming unit (IFU) was measured at 30 hpi in Wt and *ATG9A*-KO HeLa cells. The results from three independent experiments are shown as open circles. Bar graphs show the average. (**B**) Infectious progeny (IFU output/input) was calculated in Wt and *ATG9A*-KO HeLa cells at 30 hpi and expressed as a relative value. The results from three independent experiments are shown as open circles. Bar graphs show the average. Statistical significance was determined using a two-tailed Welch’s *t*-test and shown as *P*-values. (**C and D**) Generation of *ATG9*A-KO cells re-expressing *ATG9A*. Wt and *ATG9A*-KO HeLa cells and *ATG9A*-KO HeLa cells re-expressing *ATG9A* (rescue-1 and rescue-2) were fixed for immunofluorescence (**C**) or lysed for Western blot (**D**). In immunofluorescence, cells were stained with anti-ATG9A antibody (white) and Hoechst (blue). Bar: 10 µm. In Western blot, anti-ATG9A antibody and anti-GAPDH antibody as an internal control were used. A square bracket on the left indicates ATG9A. The molecular weight is labeled on the right (kDa). (**E**) Infectious progeny (IFU output/input) was calculated in Wt HeLa cells, *ATG9A*-KO HeLa cells, and rescue-1 and rescue-2 at 30 hpi and expressed as a relative value. The results from four independent experiments are shown as open circles. Bar graphs show the average. Statistical significance was determined using a two-tailed Welch’s *t*-test and shown as *P*-values or NS (not significant).

**Fig 2:**
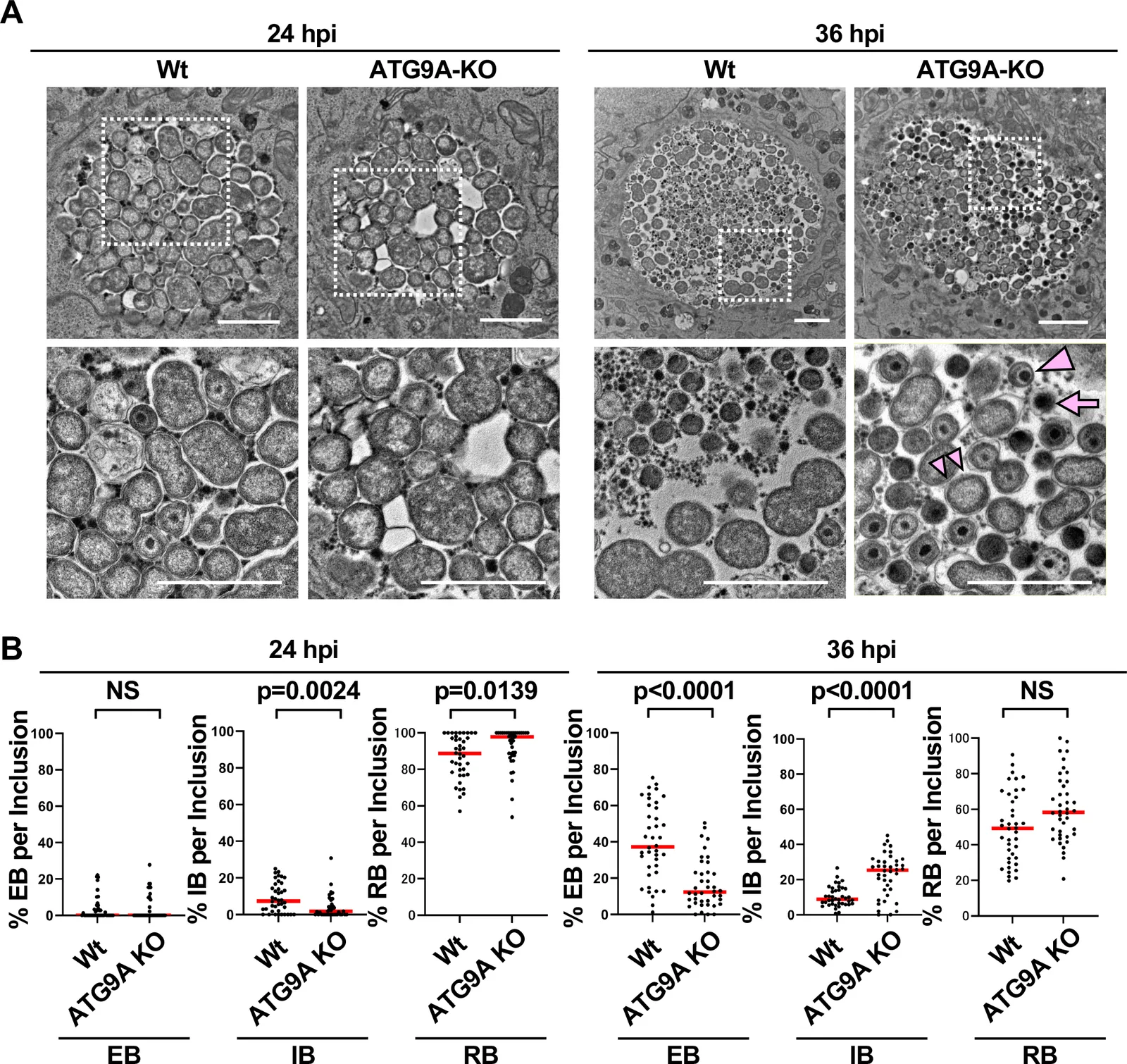
Development from RB to EB in the inclusion is delayed in *ATG9A-*KO cells. (**A**) Electron micrographs of Wt and *ATG9A*-KO at 24 and 36 hpi. The boxed regions are enlarged and shown below. The representative morphologies of EB (arrow), RB (double arrowheads), and IB (arrowhead) are shown in the image of *ATG9A*-KO at 36 hpi. Bar: 2 µm. (**B**) Quantification of EB, RB, and IB in Wt, and *ATG9A*-KO HeLa cells after infection with *C. trachomatis*. The three morphological forms were counted in 40 inclusions at 24 and 36 hpi and expressed as a percentage of the total number of bacteria in the inclusion. The results from four independent experiments are shown as a dot plot. The red line indicates the median. Statistical significance was determined using a two-tailed Mann–Whitney *U*-test and shown as *P*-value or NS (not significant).

To elucidate the importance of ATG9A in *Chlamydia* infection, we employed an *ATG9A*-KO HeLa cell line, in which autophagic flux was blocked (25). When *ATG9A*-KO and wild-type (Wt) HeLa cells were infected with *C. trachomatis* serovar L2, the infection efficiency (ratio of cells containing the inclusion) was similar to that in Wt cells (Fig. 1A). However, a significant reduction in the infectious progeny of approximately 60% was observed in the *ATG9A*-KO cells compared with Wt cells (Fig. 1B). Importantly, re-expression of *ATG9A* in *ATG9A*-KO cells (rescue-1 and rescue-2; Fig. 1C and D) restored the infectious progeny (Fig. 1E). Since only the rescued cells showing higher ATG9A levels than Wt cells were obtained, we generated Wt cells overexpressing ATG9A, which produced infectious progeny at similar levels to Wt cells (Fig. S1). This result suggests that ATG9A overexpression above control levels does not necessarily promote the microbe proliferation. Next, we examined the morphological forms of *C. trachomatis* in the inclusions using electron microscopy. At 24 hpi, the percentages of RB and IB in *ATG9A*-KO cells were, respectively, higher and lower than in Wt cells, while at 36 hpi, the percentages of IB and EB in *ATG9A*-KO cells were, respectively, higher and lower than in Wt cells (Fig. 2). These results suggest that secondary differentiation from RB to EB in the inclusions is delayed in *ATG9A*-KO cells. Taken together, we conclude that ATG9A supports *Chlamydia* growth in the inclusions.

### ATG9A supports *C. trachomatis* growth via its autophagy-independent function

**Fig 3:**
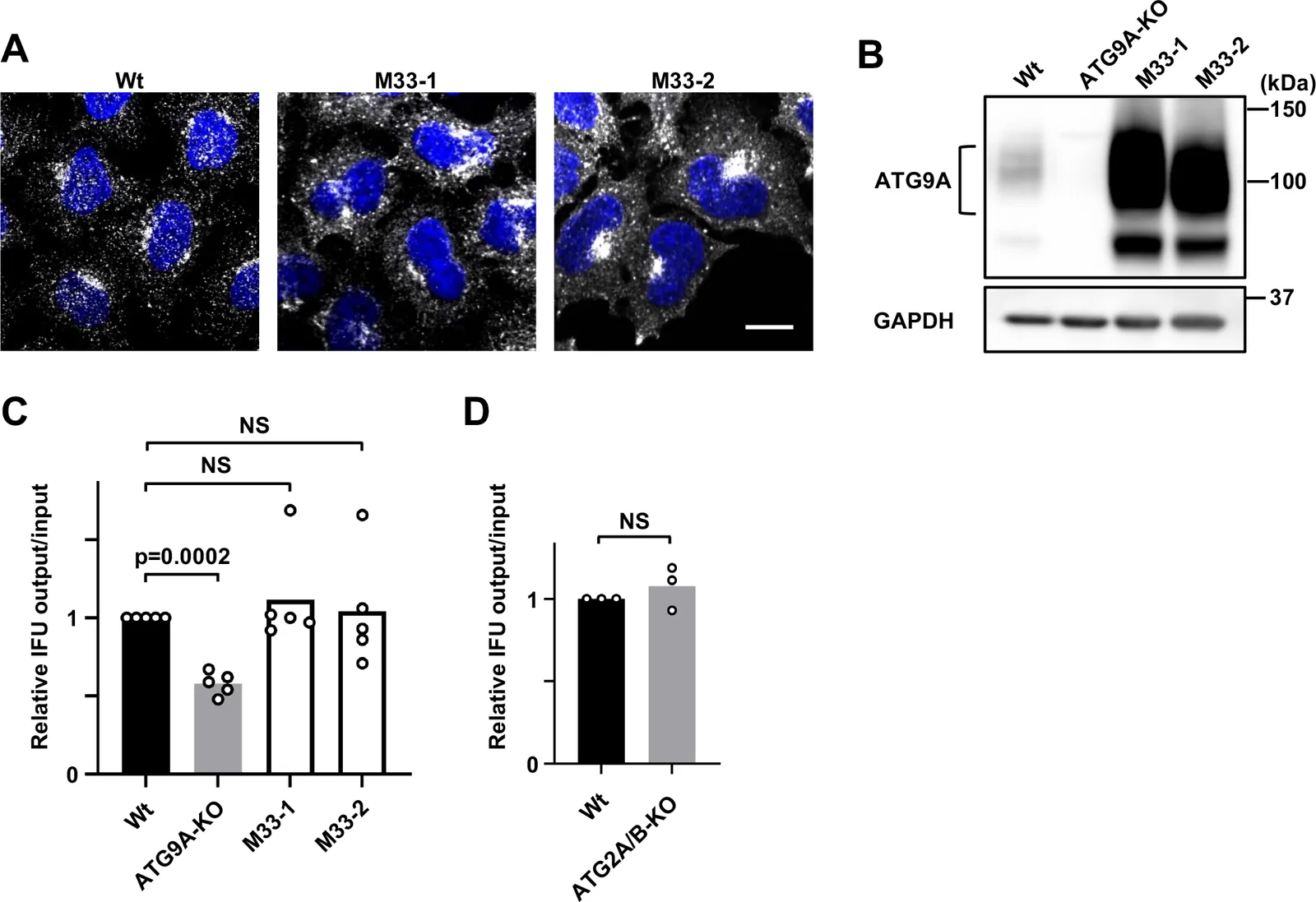
ATG9A supports *C. trachomatis* growth via its autophagy-independent function. (**A**) Wt and two clones of *ATG9A*-KO HeLa cells re-expressing *ATG9A* mutant with low scramblase activity (M33-1 and M33-2) were fixed for immunofluorescence and stained with anti-ATG9A antibody (white) and Hoechst (blue). Bar: 10 µm. (**B**) Wt, *ATG9A*-KO, M33-1, and M33-2 HeLa cells were lysed for Western blot using anti-ATG9A antibody and anti-GAPDH antibody as an internal control. A square bracket on the left indicates ATG9A. The molecular weight is labeled on the right (kDa). (**C**) The infectious progeny (IFU output/input) was calculated in *ATG9A*-KO, M33-1, and M33-2 HeLa cells at 30 hpi and expressed as a relative value. The results from five independent experiments are shown as open circles. Bar graphs show the average. Statistical significance was determined using a two-tailed Welch’s *t*-test and shown as *P*-values or NS (not significant). (**D**) The infectious progeny (IFU output/input) was calculated in Wt and *ATG2A/B*-KO HeLa cells at 30 hpi and expressed as a relative value. The results from three independent experiments are shown as open circles. Bar graphs show the average. Statistical significance was determined using a two-tailed Welch’s *t*-test. NS, not significant.

Next, we investigated whether the scramblase activity of ATG9A is required for *C. trachomatis* growth. To this end, the *ATG9A* mutant M33, which has low scramblase activity and, thus, impairs autophagy (26), was stably expressed in *ATG9A*-KO HeLa cells, and two clones were analyzed (Fig. 3A and B). The clones were confirmed to partially rescue the p62 accumulation phenotype, suggesting a partial impairment of autophagy. The infectious progeny was restored by the re-expression of the mutants (Fig. 3C), and the level of infectious progeny was comparable to that observed in *ATG9A*-overexpressing Wt cells (Fig. S1), suggesting that the autophagic function of ATG9A is not required for *Chlamydia* proliferation.

Because ATG9 has been proposed to function jointly with ATG2 in isolation membrane expansion during autophagosome formation (26 – 28), we generated *ATG2A/B* double KO HeLa cells to test whether *ATG2* deficiency shows similar effects. Autophagy flux was confirmed to be blocked in *ATG2A/B* double KO HeLa cells, whereas it was intact in *ATG2A-* or *ATG2B*-single KO cells or parental HeLa cells (Fig. S3). As a result, *ATG2* deficiency did not show significant effects on the infectious progeny (Fig. 3D). These results suggest that the lipid-supplying mechanism for autophagosome formation is not required for *C. trachomatis* growth. Therefore, we conclude that ATG9A exerts its *Chlamydia* growth-supporting function through autophagy-independent mechanisms.

### ATG9A regulates Golgi redistribution during *C. trachomatis* infection

**Fig 4:**
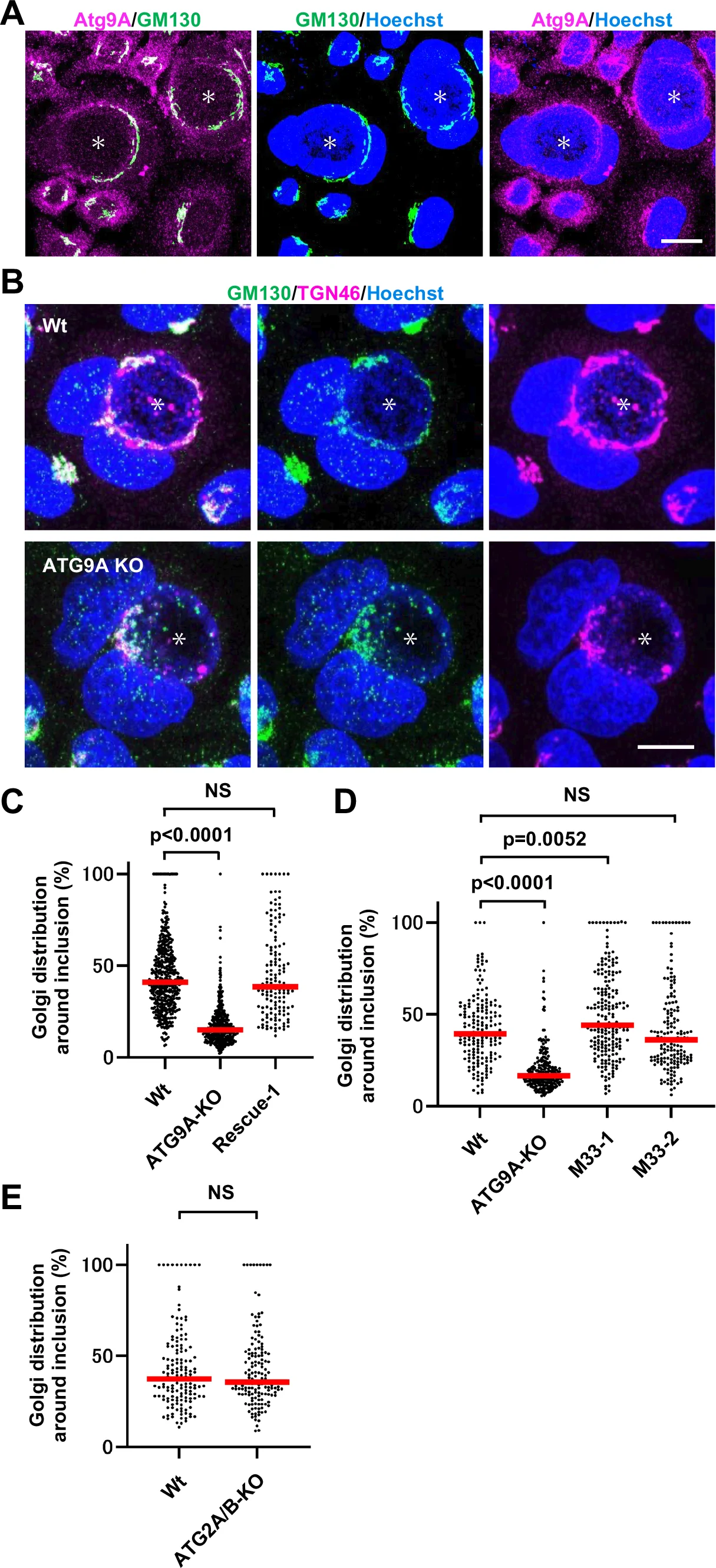
ATG9A regulates Golgi redistribution during *C. trachomatis* infection (**A**) Wt HeLa cells were infected with *C. trachomatis* and fixed at 36 hpi for double immunofluorescence with anti-GM130 antibody (*cis*-Golgi marker; green) and anti-ATG9A (magenta). They were also stained with Hoechst (DNA; blue). Images with maximum projections are shown. Asterisk: inclusion. Bar: 10 µm. (**B**) Wt and *ATG9A*-KO HeLa cells were infected with *C. trachomatis* and fixed at 36 hpi for double immunofluorescence with anti-GM130 (green) and anti-TGN46 (trans-Golgi marker; magenta) antibodies. They were also stained with Hoechst (DNA; blue). Images with maximum projections are shown. Asterisk: inclusion. Bar: 10 µm. (C–E) The degree of Golgi distribution around the chlamydial inclusion was quantified in HeLa cells as indicated. Data were expressed as a percentage of the length of the Golgi signal around the inclusion (GM130 [C] or TGN46 [D and E]) relative to the circumference of the inclusion (Hoechst). The results from three independent experiments are shown as dots. Red lines indicate median. Statistical significance was determined using a two-tailed Mann–Whitney *U*-test and shown as *P*-values or NS (not significant).

During *Chlamydia* infection, the Golgi apparatus fragments into several mini-stacked Golgi structures, which then surround the inclusion and support nutrient acquisition (7 – 9). As ATG9A is localized in the Golgi, endosomes, and plasma membrane (29 – 32), we examined whether ATG9A is relevant in the infection-induced Golgi redistribution. At 36 h after *Chlamydia* infection, GM130 (*cis*-Golgi marker) was redistributed around the inclusion, where ATG9 was colocalized (Fig. 4A). Interestingly, such alteration was not observed in *ATG9A*-KO cells (Fig. 4B). TGN46, a trans-Golgi marker, also showed the same redistribution after the infection, which was suppressed in *ATG9A*-KO cells (Fig. 4B). When the extent of Golgi marker distribution around the inclusion was measured, it was significantly lower in *ATG9A*-KO cells than in Wt cells (Fig. 4C). Moreover, the decrease was recovered by re-expressing *ATG9A* (Fig. 4C) or the mutant M33 with low scramblase activity (Fig. 4D). The levels of redistribution were similar in Wt cells, *ATG9A*-overexpressing Wt cells, and M33-overexpressing Wt cells (Fig. S1), suggesting that the overexpression does not significantly modify infection-associated Golgi redistribution. As expected, Golgi redistribution was not impaired in *ATG2A/B* double KO cells (Fig. 4E). These results suggest that ATG9A regulates Golgi redistribution during *Chlamydia* infection via autophagy-independent function.

**Fig 5:**
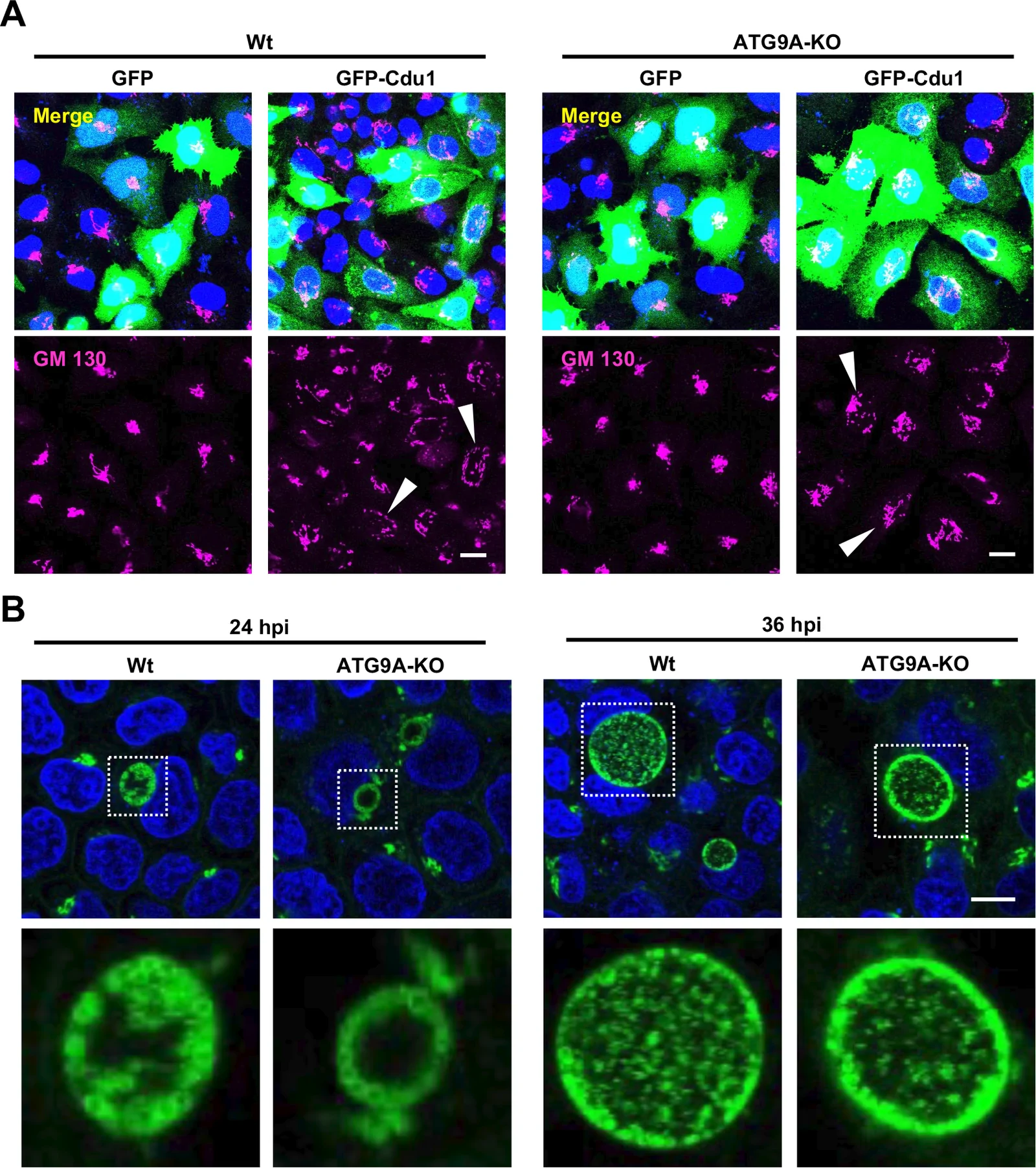
ATG9A is involved in the movement of mini Golgi stacks to the inclusion but not required for the acquisition of SM. (**A**) Wt and *ATG9A*-KO HeLa cells were transiently transfected with GFP alone or GFP-Cdu1 (green) and fixed for immunofluorescence with anti-GM130 antibody (magenta) and Hoechst staining (blue). Images for GM130 alone are shown below. Arrowheads indicate the representatives of fragmented Golgi, which is induced by the expression of GFP-Cdu1. Bar: 10 µm. (**B**) Wt and *ATG9A*-KO HeLa cells were infected with *C. trachomatis*, and BODIPY FL C5-ceramide was added at 23 hpi. Ceramide and its metabolites (green) were then imaged with confocal microscopy at 24 and 36 hpi. Boxed areas are enlarged and shown below. Note that *C. trachomatis* inside the inclusion can be detected. Blue: DNA stained with Hoechst, Bar: 10 µm.

The Golgi redistribution process is assumed to be divided into two steps: Golgi fragmentation and movement to the inclusion. Therefore, we investigated whether ATG9A participates in Golgi fragmentation, which has been reported to occur when Cdu1, a *Chlamydia* protein, is exogenously expressed in culture cells without infection (7). In Wt cells, the Golgi labeled with GM130 was concentrated in the perinuclear region in control cells (expressing GFP alone), whereas it was fragmented into small puncta around the nucleus in GFP-Cdu1-expressing cells (Fig. 5A). Such Golgi fragmentation was also observed in *ATG9A*-KO cells though it was partial (Fig. 5A). This observation suggests that ATG9A is involved in the movement of mini Golgi stacks to the inclusion rather than in the Golgi fragmentation during *Chlamydia* infection.

Because sphingolipid availability has been associated with infection-induced Golgi redistribution (9, 33), we investigated ceramide acquisition during the infection in *ATG9A*-KO and Wt cells. The fluorescence signal for BODIPY FL C_5_ ceramide is distinctly detected inside the inclusions at 24 and 36 hpi (Fig. 5B), indicating that the growth defect observed in *ATG9A*-KO HeLa cells is not caused by low acquisition of SM.

### ATG9A-N-terminal region including AP-binding motifs is required for Golgi redistribution and *C. trachomatis* growth

**Fig 6:**
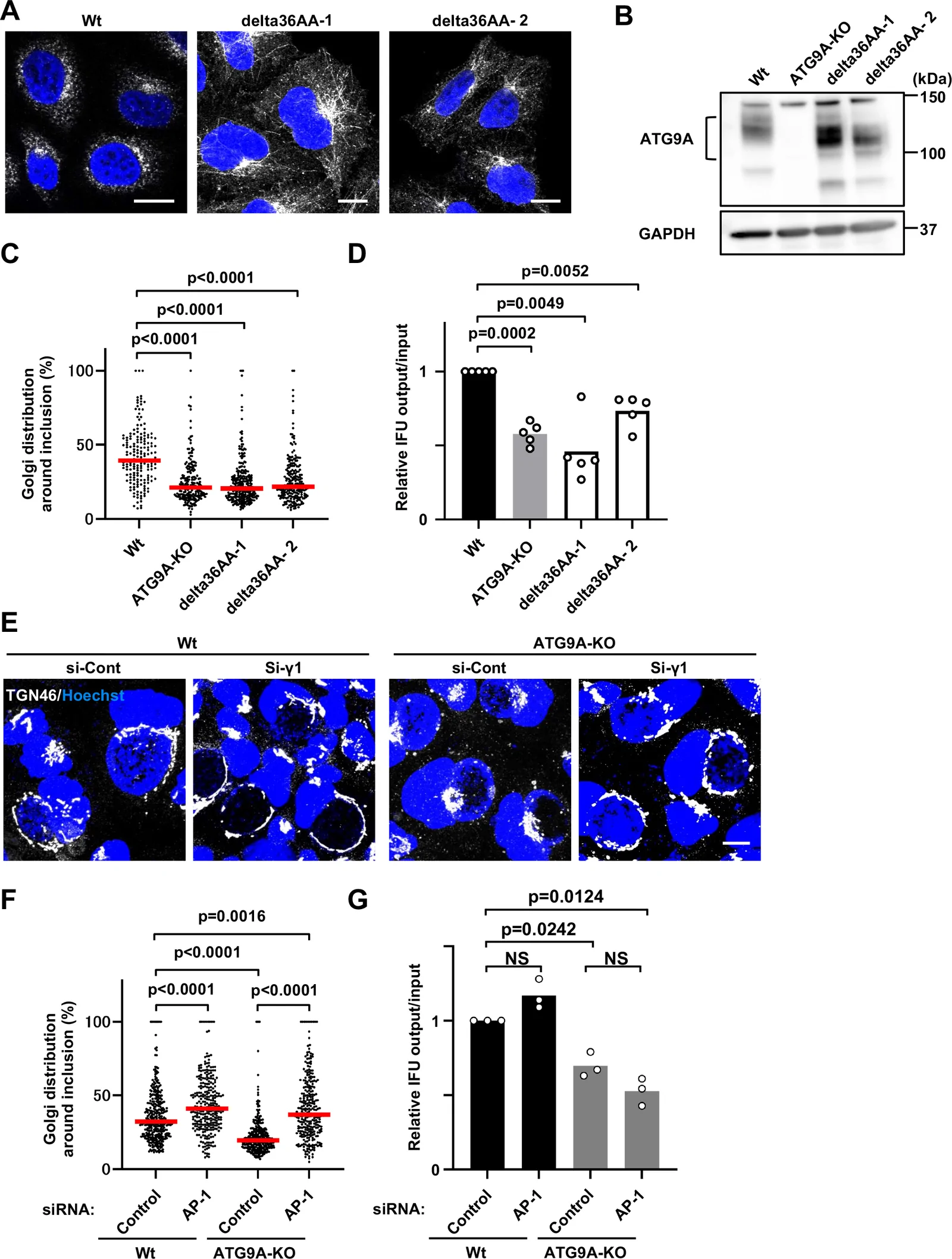
ATG9A-N-terminal region is required for Golgi redistribution and *C. trachomatis* growth. (**A**) Wt and two clones of *ATG9A*-KO HeLa cells re-expressing *ATG9A* mutant lacking N-terminal 36 amino acids (delta36AA-1 and delta36AA-2) were fixed for immunofluorescence and stained with anti-ATG9A antibody (white) and Hoechst (blue). Bar: 10 µm. (**B**) Wt, *ATG9A*-KO, delta36AA-1, and delta36AA-2 HeLa cells were lysed for Western blot using anti-ATG9A antibody and anti-GAPDH antibody as an internal control. The molecular weight is labeled on the right (kDa). (**C**) The degree of Golgi distribution around the chlamydial inclusion was quantified in *ATG9A*-KO, delta36AA-1, and delta36AA-2 HeLa cells. Data were expressed as a percentage of the length of the Golgi signal around the inclusion (TGN46) relative to the circumference of the inclusion (Hoechst). The results from two independent experiments are shown as dots. Red lines indicate median. Statistical significance was determined using a two-tailed Mann–Whitney *U*-test and shown as *P*-value or NS (not significant). (**D**) The infectious progeny (IFU output/input) was calculated in *ATG9A*-KO, delta36AA-1, and delta36AA-2 HeLa cells at 30 hpi and is expressed as a relative value. The results from five independent experiments are shown as open circles. Bar graphs show the average. Statistical significance was determined using a two-tailed Welch’s *t*-test and shown as *P*-values. (**E**) Wt and *ATG9A*-KO HeLa cells were transfected with control siRNA or siRNA for AP-1 (γ1-adaptin) and then infected with *C. trachomatis*. They were fixed at 36 hpi for immunofluorescence using anti-TGN46 (white) and Hoechst (blue). Images with maximum projections are shown. Bar: 10 µm. (**F**) The degree of Golgi distribution around the chlamydial inclusion was quantified in these cells and shown on the right. Data were expressed as a percentage of the length of the Golgi signal around the inclusion (TGN46) relative to the circumference of the inclusion (Hoechst). The results from three independent experiments are shown as dots. Red lines indicate median. Statistical significance was determined using a two-tailed Mann–Whitney *U*-test and shown as *P*-values. (**G**) The infectious progeny (IFU output/input) was calculated in the cells described in (**A**) at 30 hpi and is expressed as a relative value. The results from three independent experiments are shown as open circles. Bar graphs show the average. Statistical significance was determined using a two-tailed Welch’s *t*-test and shown as *P*-values or NS (not significant).

The N-terminal region of ATG9A binds clathrin AP complexes, AP-1, 2, and 4, which can regulate the intracellular transport of ATG9A vesicles (31, 34 – 38). Therefore, we determined whether these interactions could affect *Chlamydia* infection. An N-terminus-deleted mutant of *ATG9A* (delta36AA) was stably expressed in *ATG9A*-KO cells, and two clones were analyzed (Fig. 6A and B). The delta36AA mutant was observed as scattered cytoplasmic puncta and aberrant linear structures extended from the perinuclear portion (Fig. 6A). The delta36AA contained complexed carbohydrate chains (Fig. S4), suggesting that this mutant can exit from the ER. Neither Golgi redistribution nor *Chlamydia* growth during *C. trachomatis* infection was restored in these clones (Fig. 6C and D). These results suggest that APs regulate the proper transport of ATG9A vesicles and that this mechanism supports Golgi redistribution and *C. trachomatis* growth in the inclusion.

Given that five APs are present in humans, we investigated which APs are important for Golgi redistribution and *C. trachomatis* growth. In preliminary analysis with single siRNA experiment, knockdown (KD) of any AP did not suppress Golgi redistribution during *C. trachomatis* infection (Fig. S5). Interestingly, when the same siRNA was applied to *ATG9A*-KO HeLa cells, only AP-1 KD showed the restoration of Golgi redistribution (Fig. S5). Intensive analyses with three experiments revealed that the depletion of AP-1 increased the extent of Golgi redistribution in *ATG9A*-KO and Wt cells (Fig. 6E and F), suggesting that AP-1 restricts Golgi redistribution, and this function is enhanced by *ATG9A*-KO. Using these cells, we determined whether the redistributed Golgi components lacking *ATG9A* and AP-1 could restore *C. trachomatis* growth. As a result, low levels of infectious progeny in *ATG9A*-KO cells were not rescued (Fig. 6G), suggesting that the arrangement of ATG9A vesicles but not the other Golgi components around the inclusion is essential to facilitate *C. trachomatis* growth.

### STING-mediated interferon response is very weak in *ATG9A*-KO cells

**Fig 7:**
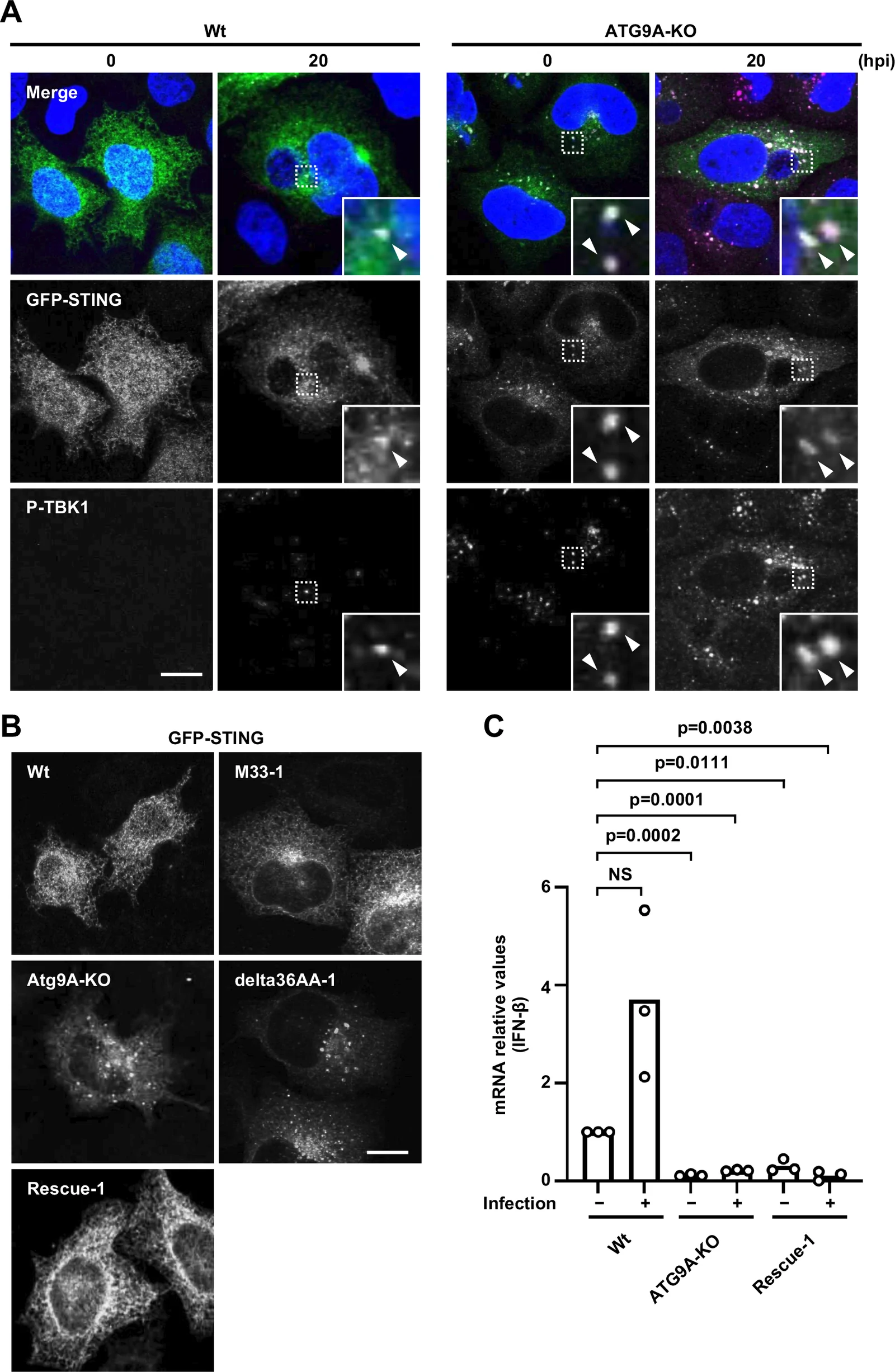
STING-mediated IFN response is very weak in ATG9A-KO cells. (**A**) Wt and *ATG9A*-KO cells were transiently transfected with GFP-STING (green) and then infected with (20 hpi) or without (0 hpi) *C. trachomatis* at an multiplicity of infection (MOI) of 5. They were fixed at 20 hpi for immunofluorescence with anti-PhosphoTBK1 antibody (magenta) and Hoechst staining (blue). Boxed areas are enlarged and shown in the insets. Arrowheads indicate colocalization of GFP-STING and phosphoTBK1. Asterisks show inclusions. Bar: 10 µm. (**B**) HeLa cells as indicated were transiently transfected with GFP-STING. Bar: 10 µm. (**C**) Wt, *ATG9A*-KO, and Rescue-1 HeLa cells were infected with (+) or without (−) *C. trachomatis*. They were lysed for mRNA extraction at 36 hpi, which were subjected to RT-qPCR for IFN-β. Data are expressed as relative values to uninfected Wt HeLa cells. Statistical significance was determined using a two-tailed Welch’s *t*-test and shown as *P*-values or NS (not significant).

A previous study demonstrated that double-stranded DNA-induced STING signaling is activated in *Atg9A*-KO mouse embryonic fibroblasts (MEFs) (22). Because this mechanism may have caused the suppression of *C. trachomatis* infection in *ATG9A*-KO HeLa cells, we examined the STING-mediated interferon (IFN) response. In Wt HeLa cells, STING was distributed with the ER pattern without *Chlamydia* infection. Moreover, at 20 hpi, the signal slightly increased in the perinuclear Golgi region. When phosphorylated TBK1 (pTBK1), a downstream molecule of STING signaling, was examined, it appeared as puncta in the perinuclear region at 20 hpi and colocalized with STING (Fig. 7A). On the other hand, STING/pTBK1-double positive puncta were distinctly observed in *ATG9A*-KO HeLa cells regardless of *Chlamydia* infection (Fig. 7A). These puncta disappeared by re-expressing ATG9A or the M33 mutant but not the delta36AA mutant (Fig. 7B), suggesting that the aberrant trafficking of ATG9A is involved in STING activation in HeLa cells. However, the gene expression of IFN-β in *ATG9A*-KO cells was significantly lower than in Wt cells irrespective of the infection, and *ATG9A* re-expression did not restore IFN-β expression (Fig. 7C), the reason for which cannot be explained at present. Nevertheless, this result suggests that ATG9A does not support *Chlamydia* growth by suppressing type I IFN response.

## DISCUSSION

ATG9A plays diverse roles that are not related to autophagy. In this study, we demonstrated for the first time that ATG9A promotes *C. trachomatis* infection. Importantly, this function is not mediated by autophagy but related to the proper arrangement of ATG9A vesicles around the inclusion. Because the defect of *Atg9A* reportedly upregulates STING-mediated type I IFN response in MEF (22), the same response may have occurred in *ATG9A*-KO HeLa cells, preventing *Chlamydia* infection (39). However, IFN-β was not significantly increased in *ATG9A*-KO HeLa cells, excluding this possibility. Although the upregulation mechanisms of STING signaling in *ATG9A*-KO MEF remain unknown (22), we speculate that the infection-induced STING signaling under *ATG9A* deficiency is regulated differently between human and mouse cells. In fact, the recognition of the inclusion by host cell defense mechanisms is known to be different between the two species (40, 41).

The molecular mechanism behind the function of ATG9A in *C. trachomatis* infection remains elusive. A role of ATG9A trafficking during the infection was inferred from the observation that delta36AA did not restore either Golgi-redistribution or infectious progeny. The N-terminal region of ATG9A can bind AP-2 and AP-4 via tyrosine-base and/or di-leucine-based motifs (35 – 38), and phosphorylation at Tyr8 in the tyrosine-based motif and Ser14 by Src and Ulk1, respectively, enhance the interaction of ATG9A with AP-1/AP-2 (38). Therefore, AP-1, AP-2, and AP-4 seem to be important in ATG9 trafficking during *C. trachomatis* infection although the siRNA experiment carried out in the present study did not identify responsible AP. In addition, *in vitro* experiments have recently shown that ATG13 can bind to the N-terminal cytoplasmic domain of ATG9 (42). Therefore, the interaction between ATG13 and ATG9A and kinase-mediated action of multiple APs could contribute to *C. trachomatis* infection.

The growth of the inclusion and *C. trachomatis* requires lipids, which are thought to be delivered by vesicular and non-vesicular pathways. Thus, several trafficking factors, such as Rabs, SNAREs, SNXs, ARFs, and Golgi matrix proteins for vesicular transport and CERT for non-vesicular transport, have been found to be associated with *Chlamydia* infection (1, 2). For example, the transport of sphingolipids to the inclusion is closely linked to *Chlamydia* infection-induced Golgi redistribution. Thus, the inhibition of Golgi matrix proteins (Golgin-84 and p115) or some Rab proteins (Rab6 and Rab11) inhibits Golgi redistribution and suppresses *Chlamydia* growth (9, 33). However, in our study, sphingolipid supply to *C. trachomatis* was not impaired in *ATG9A*-KO HeLa cells despite blocking Golgi redistribution (Fig. 5B), suggesting that the growth defect was not due to the impaired acquisition of sphingolipids. Sphingolipids might be acquired through contact sites between the ER and inclusion (43, 44) or synthesized via unknown mechanisms since *Chlamydia* can acquire sphingolipids in host cells lacking CERT/SMS1/SMS2 (45). A recent study highlighted the function of the TMEM41–ATG2A/B–ATG9A module for fatty acid mobilization from LDs to mitochondria (19). However, *ATG2A/B*-KO HeLa cells did not block *Chlamydia* growth (Fig. 3D). Thus, it is unlikely that impairment of fatty acid supply might be the reason for the suppression of *Chlamydia* growth in *ATG9A*-KO HeLa cells. Of note, the *ATG9A* mutants with impaired lipid scramblase activity still have some activities *in vitro* and in cultured cells [(26); ]. Therefore, although the expression of these *ATG9A* mutants could rescue *Chlamydia* growth, low levels of scramblase activity may have been enough to support the infection. Based on this idea, we infer that vesicle fusion and fission events occurring in the vicinity of the inclusion could be facilitated by the presence of ATG9A through its lipid scramblase activity (26, 27) and possibly membrane-bending ability (18). Recently, other roles of ATG9A have been reported, including regulation of actin cytoskeleton via binding to profilin and Ena in *Drosophila* (24), control of lamellipodial expansion and cell migration (46), protection from plasma membrane permeabilization (21), and regulation of JNK signaling through interaction with dTRAF2/TRAF6 (23). We do not exclude the possibility that these roles are linked to the supporting function of ATG9A in *Chlamydia* growth.

## MATERIALS AND METHODS

### Antibodies

Anti-major outer membrane protein (MOMP) (Santa Cruz Biotechnology), anti-TGN46 (BioRad), anti-GM130 (BD Transduction Laboratories), anti-ATG9A (Abcam), anti-phosphorylated TBK1 (Cell Signaling Technology), anti-p62 (GP62-C, Progen Biotechnik), anti-phosphorylated p62 (47), anti-LC3B (Cell Signaling Technology), anti-γ1 adaptin (BD Transduction Laboratories), anti-α adaptin (BD Transduction Laboratories), anti-ε adaptin (BD Transduction Laboratories), anti-δ adaptin (BD Transduction Laboratories), anti-ζ adaptin (Sigma-Aldrich), and anti-GAPDH (SantaCruz Biotechnology) antibodies were obtained from the respective suppliers.

### Plasmid and virus vectors

To generate an expression plasmid for GFP-tagged *C. trachomatis* Cdu1, a DNA fragment for Cdu1 sequence was obtained by polymerase chain reaction (PCR) using the forward primer GCTTCGAATTCTATGTTATCTCCCACCAACTCAAC, the reverse primer GGTACCGTCGACTGCTTCAGGCCAAGAAAGCTCTG, and *C. trachomatis* L2 strain434/Bu as a template. The PCR product was inserted into the EcoRI/SalI site of pEGFP-C1 (Takara Bio Inc.).

To generate plasmids containing full-length ATG9A or its N-terminal deleted mutant (delta36AA), the corresponding DNA fragments were obtained by PCR from a cDNA library of HeLa cells. The PCR products were inserted in pLVSIN-Puro vectors by using the In-Fusion system (Takara Bio Inc.). cDNA for the *ATG9A* mutant M33 was kindly provided by Dr. Takanori Otomo (The Scripps Research Institute) and cloned in pLVSIN-Puro vectors. The pMX-IPuro-EGFP-human STING plasmid vector was kindly provided by Dr. Tomohiko Taguchi (Tohoku University). The virus vector was produced in HEK293T cells by co-transfecting a pLVSIN-Puro vector containing full-length *ATG9A* or mutant *ATG9A*, a VSVG vector, a RTR2 vector, and a 4.1R vector using the FuGENE HD system (Promega).

### Cells and transfection

Cells were cultured in Dulbecco’s modified Eagle medium (NACALAI TESQUE, INC.) supplemented with 10% fetal bovine serum (Sigma-Aldrich) at 37°C in a humidified atmosphere containing 5% CO_2_. Vero (CCL-81) cells were purchased from ATCC. Wt and *ATG9A*-KO HeLa cells (25) were kindly provided by Dr. Noboru Mizushima (The University of Tokyo).

To generate *ATG2A/B* double KO HeLa cells, each guide RNA designed using the CRISPR design tool (https://crispr.dbcls.jp/) was subcloned into pX330-U6-Chimeric_BB-CBh-hSpCas9 (Addgene, 42230; deposited by Feng Zhang’s lab), a human codon-optimized SpCas9, and chimeric guide RNA expression plasmid. First, HeLa cells were co-transfected with the vectors pX330 for *ATG2A* and pEGFP-C1 (Takara Bio Inc.) and cultured for 2 days. Thereafter, GFP-positive cells were sorted and expanded. The loss of the genes was confirmed by heteroduplex mobility assays followed by immunoblot analysis with antibodies. *ATG2A/B* double KO HeLa cells were made on the basis of *ATG2A* KO HeLa cells.

To generate Wt and ATG9A-KO HeLa cells stably expressing ATG9A, M33, and delta36AA, cells were infected with virus particles in media containing 8 µg/mL polybrene for 2 days prior to selection with 5 µg/mL puromycin. A single clone was selected by limiting dilution. For experiments of transient expression, cells were transfected with plasmids using FuGENE HD and cultured for 48 h.

### Chlamydia strain and infection

*Chlamydia trachomatis* strain *L2/434/Bu* (ATCC) was propagated in Vero cells, harvested by water lysis at 48 hpi, sonicated, diluted in sucrose–phosphate–glutamate (SPG) buffer (75 g/L sucrose, 0.5 g/L KH_4_PO_4_, 1.2 g/L Na_2_HPO_4_, 0.72 g/L glutamic acid; pH 7.5), and stored at −80°C. For synchronized infection, cells were centrifuged with *C. trachomatis* at 2,500 × *g* for 30 min at 10°C in an Avanti HP-26 XP Centrifuge (Beckman).

### Infection efficiency assay

Cells were seeded in 96-well plates and infected with *C. trachomatis* by using a series of 1:10 diluted original solution. At 30 hpi, they were fixed with ice-cold methanol and stained with anti-MOMP antibody and Hoechst. Images were captured with a BZ-9000 fluorescence microscope (KEYENCE). For the quantification of inclusion-forming units (IFUs), the number of MOMP-positive inclusions was counted using Fiji software and divided by the volume of the original bacterial lysate.

### Infectious progeny assay

Infectious progeny assay was performed according to a previous study (48). Briefly, cells were seeded in two 96-well plates and infected with *C. trachomatis* at a multiplicity of infection (MOI) of 0.5. At 30 hpi, the cells in one of the plates were fixed with ice-cold methanol and stored in PBS at 4°C (input). The cells in another plate were lysed and diluted in SPG. A series of 1:10 dilutions was used to infect Wt HeLa cells in an additional 96-well plate. At 30 hpi, the cells were fixed and stained as described above (output), together with cells in the input plates. Images were captured on BZ-9000 (KEYENCE) or CQ1 system (YOKOGAWA). The IFUs of the input (input IFU) and output (output IFU) plates were quantified as described above using Fiji or CellPathfinder software (YOKOGAWA). Output IFU was normalized to its respective input IFU, which was used as the infectious progeny. The ratio to the infectious progeny of control cells was plotted in graphs. Data were obtained from at least three independent biological replicates.

### siRNA knockdown

siRNAs against the following genes were obtained from Dharmacon: human γ1-adaptin (L-019183-00-0005), α-adaptin (L-012492-00-0005), ε-adaptin (L-021474-00-0005), δ-adaptin (L-016014-00-0005), ζ-adaptin (L-025284-01-0005), and Ambion Silencer Negative Control (AM4611). Cells were transfected with these siRNA using Lipofectamine RNAiMAX (Invitrogen) according to the manufacturer’s instructions and cultured for 48 hr.

### Reverse transcription-quantitative PCR

RNA was extracted from culture cells using ISOGEN (NIPPON GENE). Gene expression levels were analyzed using the TaqMan qPCR assay kit (Thermo Fisher Scientific) and Step-One-Plus Real-Time PCR system (Thermo Fisher Scientific). Probes for IFN-β (Hs01077958 s1 IFNB1) and GAPDH (Hs02758991 g1 GAPDH) were used. Gene expression levels of IFN-β were normalized with GAPDH levels.

### Ceramide uptake

Cells were seeded in glass bottom dishes and infected with *C. trachomatis* at an MOI of 0.5. Five µM BODIPY FL-C_5_ Ceramide (Thermo Fisher Scientific) was added in the culture medium at 23 hpi for 30 min at 4°C. After removing ceramide from the culture medium, cells were incubated at 37°C up to 24 and 36 hpi.

### Immunofluorescence microscopy and quantification of Golgi redistribution

Cells were fixed with 3% paraformaldehyde in 0.1 M phosphate buffer for 15 min. After permeabilization with 0.1% Triton X-100 in PBS for 5 min or with −20°C methanol for 10 min, the cells were incubated with 3% BSA for blocking and then with primary antibodies followed by secondary antibodies conjugated with Alexa 488 and/or 594. Images were captured using a confocal microscope (FV1000, Olympus) equipped with an Apochromat 63× lens with a 1.40 numerical aperture. Fiji software (NIH) was used for data analysis.

For measurement of the degree of Golgi redistribution, maximum intensity Z-projection images were generated. The length of Golgi signal around the inclusion and perimeter of the inclusion were measured using the line tool in Fiji software. The percentage of the length of Golgi relative to that of the inclusion perimeter was calculated. Three independent experiments were performed, with 10 fields analyzed for each experiment.

### Electron microscopy

Cells cultured on coverslips were fixed with 2% formaldehyde and 2% glutaraldehyde in 0.1 M phosphate buffer (pH 7.4) and post-fixed in a mixture of 1% osmium tetroxide and 1.5% potassium ferrocyanide in the same buffer. After ethanol dehydration, samples were embedded in epoxy resin. Ultrathin sections were observed with an electron microscope (JEM1400EX, JEOL) operated at 80 kV.

### *In vivo* glycosylation and Western blot analyses

*ATG9A*-KO HeLa cells stably expressing *ATG9A* or delta36AA mutants were treated with 10 mg/mL cyclohexamide for 2 h, homogenized, and solubilized with 1% TritonX-100. The supernatant obtained by centrifugation at 15,000*g* was treated with either Endo H or PNGase F (New England Biolabs) glycosidase enzymes.

Total cell lysates were dissolved in an SDS-containing sample buffer and analyzed by Western blot. The blot signal was detected by chemiluminescence and captured with an Image Quant LAS 4000 (GE Healthcare).

### Autophagy flux assay

Cells were infected with *C. trachomatis* at an MOI of 10. Subsequently, they were treated with or without 100 nM Bafilomycin A1 (Merck Millipore) at 20 hpi for 1 h and lysed with 2% SDS buffer for Western blot analysis.

### Statistical analyses

All statistical analyses were performed using GraphPad Prism 9. Statistical significance was considered at *P* < 0.05.

## Data Availability

The data that support the findings of this study are available from the corresponding author, SW, upon reasonable request.

## SUPPLEMENTAL MATERIAL

Supplemental figures spectrum.02774-23-s0001.pdf Figures S1 to S5.

ASM does not own the copyrights to Supplemental Material that may be linked to, or accessed through, an article. The authors have granted ASM a non-exclusive, world-wide license to publish the Supplemental Material files. Please contact the corresponding author directly for reuse.
