# Supplementary material for: ATG9A supports Chlamydia trachomatis infection via autophagy-independent mechanisms
Source: Microbiol Spectr. 2023 Sep 14;11(5):e02774-23. doi: 10.1128/spectrum.02774-23 (PMC10580829; doi:10.1128/spectrum.02774-23)
Supplement: Supplemental figures — Figures S1 to S5. [file spectrum.02774-23-s0001.pdf]

Fig. S1

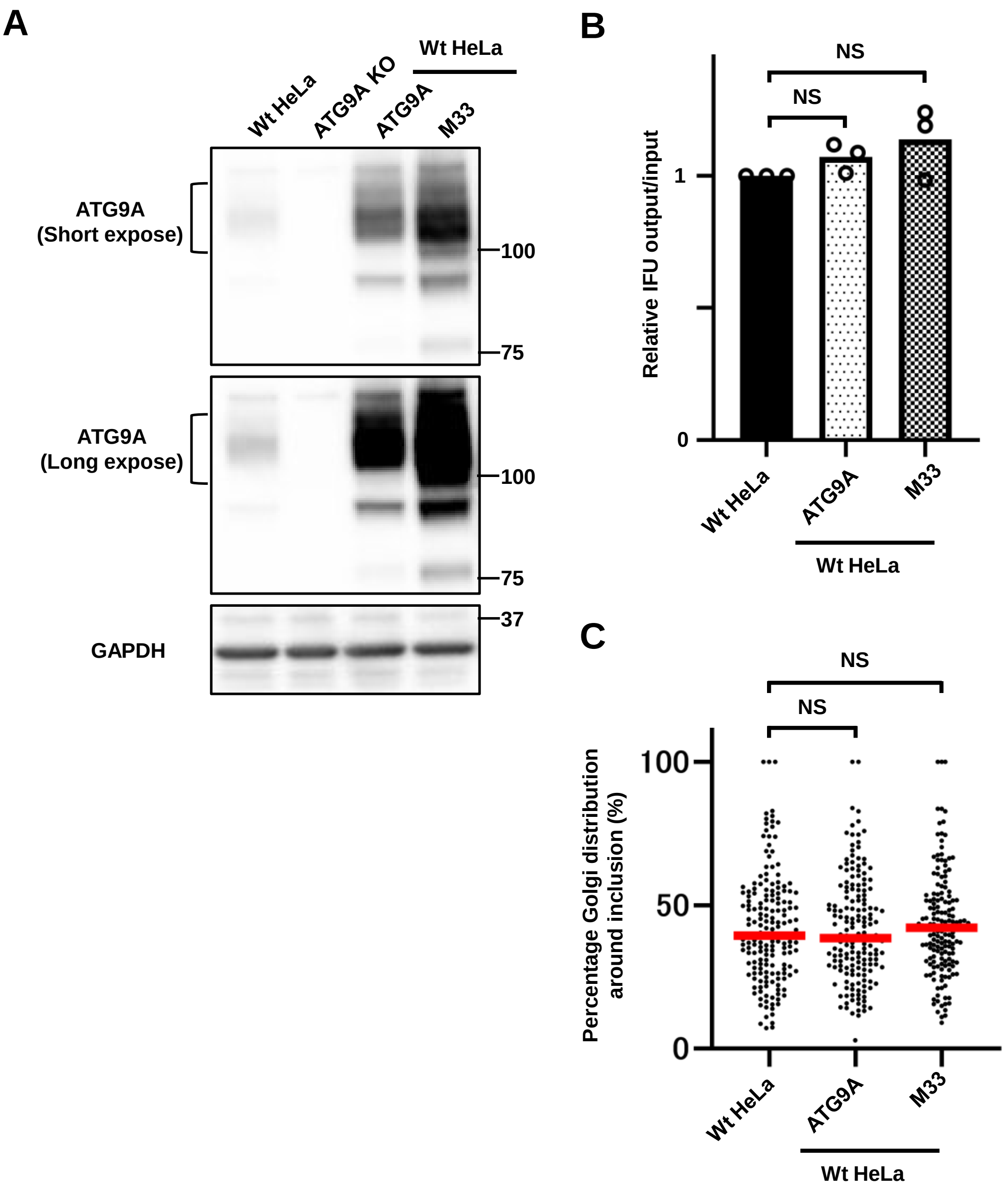

Figure S1. Overexpression of *ATG9A* or M33 mutant in Wt cells shows no significant alterations in infectious progeny and Golgi redistribution

A) Wt cells, *ATG9A*-KO cells, *ATG9A*-overexpressing Wt cells, and M33-overexpressing Wt cells were lysed for Western blot using anti-ATG9A antibody and anti-GAPDH antibody as an internal control. The square bracket on the left part shows ATG9A. The molecular weight is indicated on the right (kDa). B) At 30 hpi, the infectious progeny (IFU output/input) of Wt cells, *ATG9A*-overexpressing Wt cells, and M33-overexpressing Wt cells was calculated and expressed as a relative value. The open circles show the results from three independent experiments. Bar graphs show average values. Statistical significance was determined by the two-tailed Welch's *t*-test. NS: not significant. C) Golgi distribution around the chlamydial inclusion was quantified in Wt cells, *ATG9A*-overexpressing Wt cells, and M33-overexpressing Wt cells. Data are expressed as percentages of the length of the Golgi signal around the inclusion (TGN46) relative to its circumference (Hoechst). The dots show the results from three independent experiments. Red lines indicate median values. Statistical significance was determined by the two-tailed Mann–Whitney *U*-test. NS: not significant.

Fig. S2

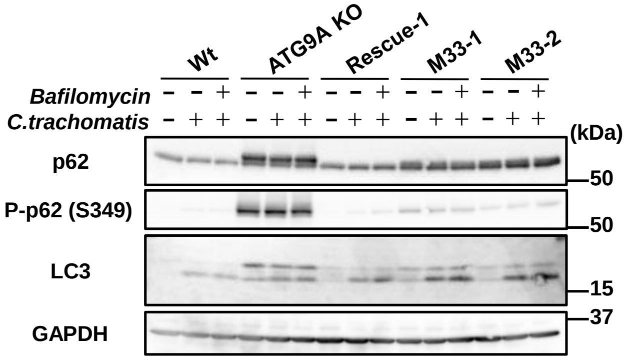

Figure S2. M33 cells partially recovered autophagic activity

Wt, *ATG9A*-KO, Rescue-1, M33-1, and M33-2 cells were infected with *C. trachomatis* at an MOI of 10. At 19 hpi, they were further incubated with or without 100 nM of bafilomycin A1 for 1 h, and lysed for Western blot using antibodies against p62, S349-phosphorylated p62, LC3, and GAPDH as an internal control. A drastic accumulation of either p62 or phosphorylated p62 (S349) was observed in *ATG9A*-KO cells, and it was partially restored in M33-1 and M33-2 cells.

Fig. S3

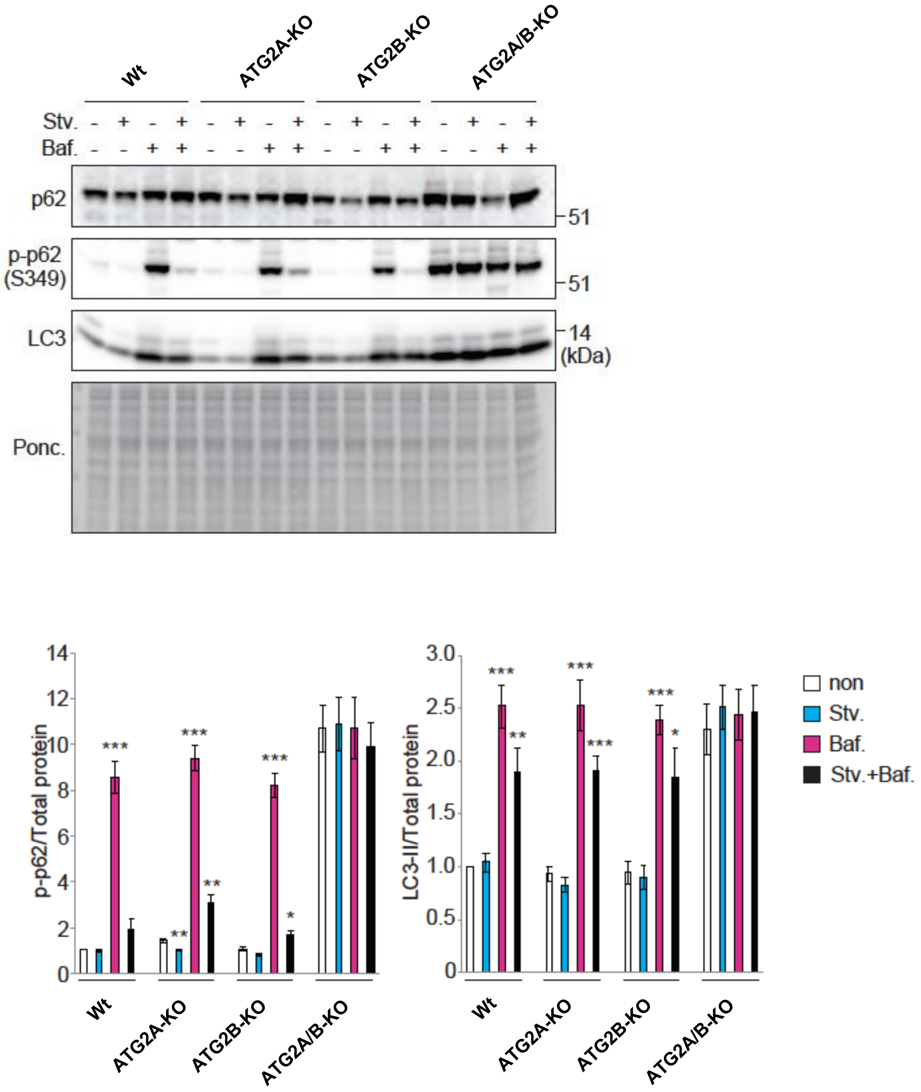

Figure S3. Autophagy flux is blocked in *ATG2A/B* double KO HeLa cells
Parental, *ATG2A*-, *ATG2B*-, and *ATG2A/B*-deficient HeLa cells were cultured in nutrient-rich or -poor conditions in the presence or absence of bafilomycin A_1 (Baf). The cell lysates were subjected to sodium dodecyl sulfate–polyacrylamide gel electrophoresis followed by Western blot analysis with the indicated antibodies and Ponceau staining. Graphs show the levels of LC3-II and S349-phosphorylated p62 per total protein estimated by Ponceau staining. Data shown are representative of four separate experiments. Data are means ± SEM. *p < 0.05, **p < 0.01, and ***p < 0.001, as determined by Welch's *t*-test.

Fig. S4

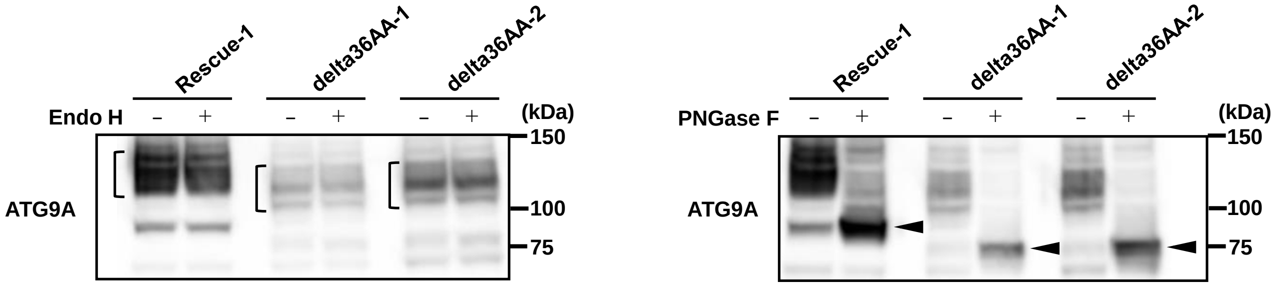

Figure S4. Delta36AA mutant can exit from the ER.
ATG9A-KO cells expressing either *ATG9A* or delta36AA were treated with 10 µg/ml cycloheximide for 2 hr. Cell lysates were prepared and treated with Endo H (left) or PNGase F (right). Note that the bands for ATG9A or delta36AA mutants (square brackets) were shifted to lower positions (arrowhead) by the treatment with PNGase F, but not with Endo H.

Fig. S5

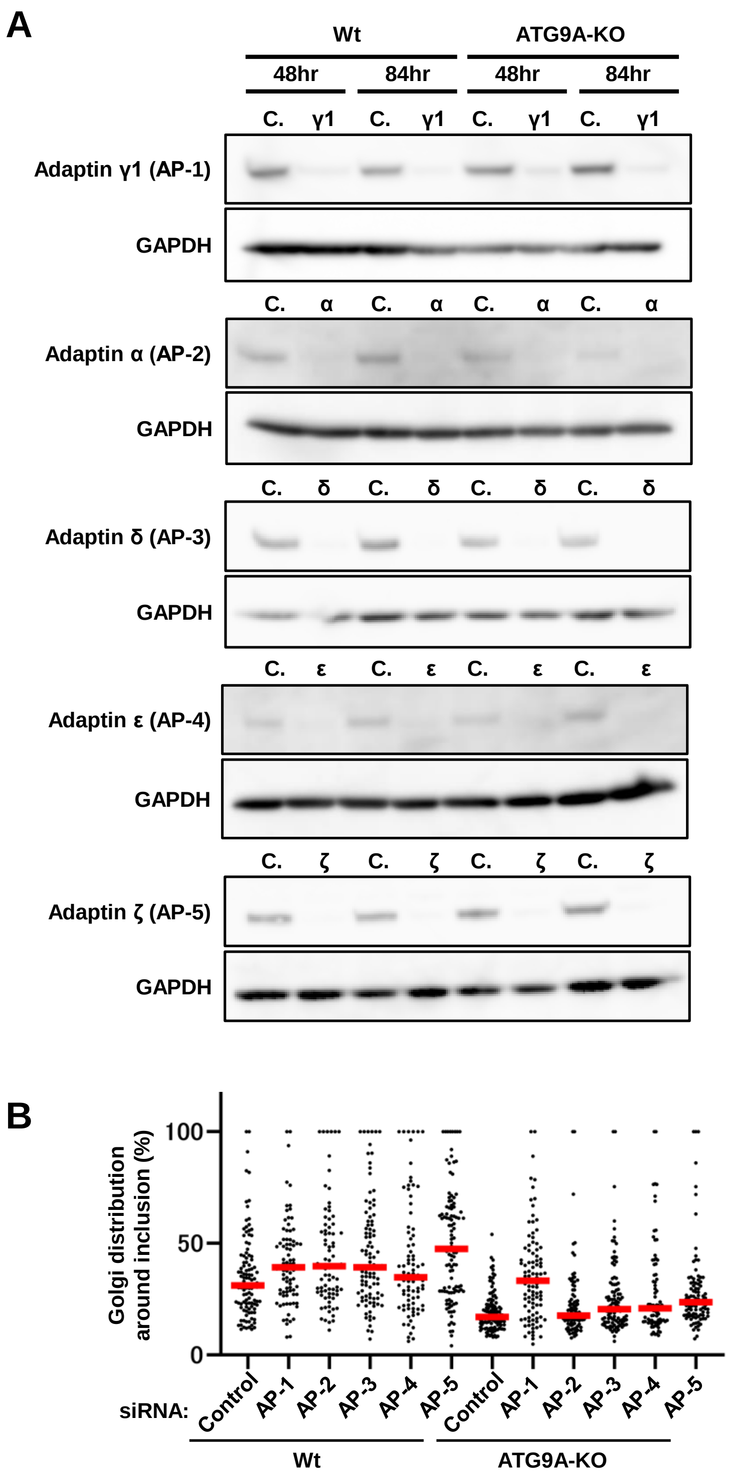

Figure S5. Influences of the depletion of five APs on Golgi redistribution

A) Wt and *ATG9A*-KO cells were transfected with control siRNA (C) or siRNA for AP-1 (γ1-adaptin), AP-2 (α-adaptin), AP-3 (δ-adaptin), AP-4 (ε-adaptin), or AP5 (ζ-adaptin). They were cultured for 48 or 84 h and lysed for Western blot using the corresponding antibodies and anti-GAPDH antibody as an internal control. B) The degree of Golgi distribution around the chlamydial inclusion was quantified in the cells described in (A). Data were expressed as a percentage of the length of the Golgi signal around the inclusion (TGN46) relative to the circumference of the inclusion (Hoechst). The results from a single experiment are shown as dots. Red lines indicate median.
